# Transcriptome sequencing combined with experimental verification to explore potential key genes related to uric acid in diabetic retinopathy

**DOI:** 10.1371/journal.pone.0350132

**Published:** 2026-06-02

**Authors:** Haiming Liang, Tianqi Yang, Feina Lu, Lixia Lin, Hao Liang

**Affiliations:** 1 Department of Ophthalmology, The First Affiliated Hospital of Guangxi Medical University, Nanning, Guangxi, China; 2 Department of Ophthalmology, Guangxi Hospital, The First Affiliated Hospital of Sun Yat-sen University, Nanning, Guangxi, China; 3 Guangxi Medical University, Nanning, ‌‌Guangxi, China; 4 Department of Ophthalmology, The First People’s Hospital of Nanning, Nanning,‌‌ Guangxi, China; Sanmenxia Central Hospital, Henan University of Science and Technilogy, CHINA

## Abstract

**Purpose:**

Diabetic retinopathy (DR), a major microvascular complication of diabetes and leading global blindness cause, involves uric acid (UA) in its onset and progression. This study aimed to identify UA-related genes (UARGs) in DR and clarify their molecular mechanisms for improved diagnosis and treatment.

**Methods:**

Using public database transcriptome data, key UARGs were screened via differential expression analysis, machine learning, receiver operating characteristic (ROC) analysis, and expression profiling, followed by gene set enrichment analysis (GSEA), immune infiltration analysis, molecular regulatory network construction, and clinical validation with reverse transcription quantitative polymerase chain reaction (RT-qPCR).

**Results:**

*MMP15* and *FOXK1* were identified as potential key genes, with significantly elevated expression in DR patient blood samples. GSEA showed *MMP15* enriched in Apc targets requiring Myc and Smarca2 targets up, and *FOXK1* in phosphatidylinositol signaling system and proteasome. Immune infiltration analysis revealed differences in 7 immune cell types, with central memory CD8 T cells and natural killer cells showing the strongest positive correlation; *MMP15* was negatively correlated with NK cells, and *FOXK1* with central memory CD4 T cells and effector memory CD8 T cells. Twelve transcription factors (e.g., HOXB7, CATA6) jointly targeted both genes.

**Conclusion:**

*MMP15* and *FOXK1* were identified as potential key genes associated with uric acid in DR, which may provide a reference for further exploration of the pathogenesis and targeted therapy of DR.

## 1. Introduction

Diabetic retinopathy (DR) is one of the most common microvascular complications of diabetes mellitus and a leading cause of vision loss worldwide. Its pathogenesis is driven by chronic hyperglycemia and involves complex processes such as microvascular damage and pathological neovascularization [1,2]. With the continuously increasing prevalence of diabetes globally, DR has become the primary cause of acquired blindness in the working-age population [2,3]. Although current therapeutic approaches, including anti-vascular endothelial growth factor (VEGF) agents, vitrectomy, and panretinal photocoagulation, have been applied clinically, they still have limitations such as the requirement for repeated injections, poor treatment compliance, and unsatisfactory responses in some patients [4–7]. Therefore, in-depth exploration of the key molecular pathways underlying the pathogenesis of DR is crucial for the development of more precise and effective therapeutic strategies.

Uric acid (UA) is the end product of purine metabolism. In addition to serving as a diagnostic marker for gout, epidemiological studies have suggested that elevated UA levels are associated with an increased risk of diabetes mellitus and its complications, including diabetic nephropathy and peripheral neuropathy [3]. However, current epidemiological evidence regarding the relationship between UA and DR remains highly inconsistent. Several studies have demonstrated that high UA levels are an independent risk factor for DR and correlate with the severity of DR [8,9], whereas other studies have found no independent association between the two, and even reported contradictory results [10]. Such inconsistency indicates that the mechanism underlying the role of UA in DR pathogenesis may be highly complex, rather than a simple linear correlation.

Mechanistic studies have shown that uric acid can trigger the release of inflammatory mediators, including tumor necrosis factor-α (TNF-α), interleukin-6 (IL-6), and C-reactive protein (CRP) [11], which subsequently promote the recruitment and activation of immune cells, thereby establishing a self-sustaining inflammatory amplification cascade. Consistent with this, recent meta-analyses have indicated that elevated IL-6 levels are associated with an increased risk of DR [12]. Furthermore, in vitro studies have revealed that under high-glucose conditions, increased uric acid levels promote the expression of inflammatory mediators and enhance the activity of the Notch signaling pathway in retinal endothelial cells [13]. In addition, hyperuricemia induces endothelial dysfunction by impairing endothelial cell function and upregulating vascular endothelial growth factor (VEGF) expression [14,15]. Nevertheless, the precise role of uric acid in DR pathogenesis remains elusive due to inconsistent epidemiological evidence and a lack of systematic bioinformatics investigations. Therefore, the present study aims to elucidate the potential key genes and regulatory pathways linking uric acid to DR through integrated analysis, so as to provide new insights into the association between UA and DR and identify potential molecular targets for targeted therapy of DR.

In this study, we leveraged publicly available transcriptome datasets to identify UA-related hub genes in DR through differential expression analysis, machine-learning-based feature selection, and expression validation. We then performed gene set enrichment analysis (GSEA), immune cell infiltration profiling, and GeneMANIA-based functional association analysis, and further constructed putative regulatory networks around these genes. Our goal was to elucidate plausible molecular mechanisms linking UA-associated inflammatory and innate immune activity to retinal vascular pathology in DR, and to provide a theoretical foundation for future translational and clinical studies.

## 2. Materials and methods

### 2.1. Data collection

Gene expression information of the GSE221521 and GSE185011 datasets related to DR was retrieved from the Gene Expression Omnibus (GEO). Specifically, GSE221521 (training set) (platform: GPL24676) included 69 DR patient samples and 50 control blood samples [1]. GSE185011 (validation set) (platform: GPL24676) included 5 DR patient samples and 5 control blood samples [16]. Both datasets were processed using official standardized pipelines. Specifically, the GEO official pipeline utilized HISAT2 to align reads to the GRCh38/hg38 reference genome, and featureCounts was used to obtain raw counts at the gene level. The raw counts were then normalized using the FPKM (Fragments Per Kilobase of transcript per Million mapped fragments) method: FPKM = C × 10⁹/ (N × L), where C represented the number of fragments for a gene, N represented the total number of aligned fragments (sequencing depth) for the sample, and L represented the total length of exons (bp) for the gene. Both datasets were processed using the same normalization pipeline, and the official FPKM matrix files were directly downloaded for subsequent analyses. Detailed clinical information for both datasets is provided in **S1 Table**. In addition, for independent external validation, a new dataset, GSE94019 (platform: GPL11154), which included 9 DR patient samples and 4 control samples, was introduced. For literature reference, the keyword “Uric Acid” was searched in the GeneCards database, and 3,806 uric acid-related genes (UARGs) were obtained [11] (**S2 Table**).

### 2.2. Differential expression analysis

In the GSE221521 dataset, differential expression analysis between DR patients and controls was performed using the DESeq2 package (v 1.40.2) [17] with thresholds of p < 0.05 and |log₂FC| > 0.5. The top 10 up- and down-regulated genes, ranked by log₂FC values, were visualized in a volcano plot via the EnhancedVolcano package (v 1.18.0) (https://github.com/kevinblighe/EnhancedVolcano) and an expression heatmap using the ComplexHeatmap package (v 2.16.0) [18].

### 2.3. Identification of candidate genes

The overlap of DEGs and UARGs (candidate genes) was visualized with the Venn Diagram package (v 1.7.3) [19]. Functional annotation of candidate genes was then conducted using Gene Ontology (GO) enrichment analysis—covering BP, CC, and MF categories—and Kyoto Encyclopedia of Genes and Genomes (KEGG) pathway enrichment analysis for pathway annotation of all identified proteins. GO and KEGG enrichment analyses of candidate genes were executed via the clusterProfiler package (v 4.15.0.003) [20] (p-adjust < 0.05, Benjamini & Hochberg) with gene annotation derived from org.Hs.e.g.,db (v 3.17.0) [21]. The results were visualized via the ggplot2 package (v 3.5.1) [22]. Meanwhile, candidate genes were submitted to the STRING database to construct an interaction network (interaction score > 0.4), which was subsequently visualized in Cytoscape software (v 3.10.2) [23].

### 2.4. Identification of potential key genes

Machine learning, a robust data analysis methodology, has been extensively applied to genetic feature screening. To screen the obtained feature genes, an algorithm was used: the Least Absolute Shrinkage and Selection Operator (LASSO) regression. Based on the GSE221521 dataset, LASSO regression was executed via the glmnet package (v 4.1-8) [24], and using 10-fold cross-validation, the optimal λ value was determined to screen feature genes at the minimum error threshold.

In an effort to evaluate how feature genes differentiate DR blood samples from control ones, ROC analysis was conducted using the pROC package (v 1.18.5) [25]. on the feature genes in the DR and control blood samples of the GSE221521 dataset. The area AUC value was calculated. Feature genes (AUC > 0.7) for distinguishing DR blood samples were selected from the GSE221521 dataset, and their ROC curves were displayed.

For additional confirmation of the feature genes’ expression levels, a Wilcoxon test (p < 0.05) was used. Feature gene expression was validated across all samples from the GSE221521 and GSE185011 datasets. Using the ggplot2 package (v 3.5.1), boxplots were constructed to present the outcomes. Based on significant differential expression and coherent trends across both datasets, a core set of genes was defined for all downstream analyses. To further ensure the generalizability of the results, the cohens_d function from the R package rstatix was used to perform effect size testing on the validation dataset. The magnitude of the difference between the two groups was assessed based on the absolute value of Cohen’s d, with the following criteria: < 0.2 indicating a very small effect, 0.2-0.5 indicating a small effect, 0.5-0.8 indicating a medium effect, and ≥ 0.8 indicating a large effect.

### 2.5. GSEA

For a deeper understanding of the biological functions of potential key genes in all GSE221521 dataset samples, the reference gene set was derived from the KEGG pathway gene set c2.cp.all.v2022.1.Hs.symbols.gmt (provided by the MsigDB). The stats package (v 4.3.1) was employed for the purpose of conducting Spearman correlation analysis for each key gene against all other genes, respectively, to obtain the correlation coefficients of the genes. These coefficients were then sorted from largest to smallest, and GSEA was executed via the clusterProfiler package (v 4.15.0.003). The GSEA results were visualized with the enrichplot package (v 1.20.3) [26], applying a statistical threshold of p-adjust < 0.05 (Benjamini & Hochberg).

### 2.6. Immune infiltration analysis

Using the single-sample Gene Set Enrichment Analysis (ssGSEA) algorithm in the GSVA package (v 0.99.0) [27], the infiltration abundances of 28 immune cell types were evaluated in the GSE221521 dataset to compare DR and control blood samples [28]. Subsequently, infiltration differences of 28 immune cells between DR and control groups were assessed using the Wilcoxon test (p < 0.05) and visualized by boxplots generated with the ggplot2 package (v 3.5.1). Next, pairwise correlations involving differentially infiltrated immune cells and potential key genes across all GSE221521 samples were assessed using the Spearman method from the psych package (v 2.4.6.26) [29]. Significant correlations (|r| > 0.3, p < 0.05) were plotted using the ggplot2 package (v 3.5.1).

### 2.7. GeneMANIA analysis

The potential key genes were submitted to the GeneMANIA database (species: Homo sapiens) to construct a protein-protein interaction (PPI) network with functionally similar genes, thereby enabling functional characterization.

### 2.8. Molecular regulatory network analysis

An mRNA-TF regulatory network was established, and the transcriptional regulatory effects of transcription factors (TFs) on potential key genes were analyzed to reveal potential regulatory mechanisms. Furthermore, interaction information between TFs and their target genes was extracted from the ChIPBase database. Transcription factors with a combined count of “discovered samples (upstream)” and “discovered samples (downstream)” greater than 12 were designated as key TFs. Data was organized and filtered using the tidyverse package (v 2.0.0) [30], and construction of the regulatory network was executed using igraph, followed by visualization with Cytoscape software (v 3.10.2).

Correlation analysis between purine metabolism‑related genes and *FOXK1* was performed To investigate whether *FOXK1* exhibited co‑expression relationships with genes in the purine metabolism pathway, eight genes were selected, including the core purine synthesis enzymes (PPAT, ADSL, ATIC, IMPDH1/2), core degradation enzymes (ADA, XDH), and transporters/receptors (ENTPD1, CD39). Based on the gene expression matrix of all samples in the GSE221521 dataset, the Pearson correlation coefficients between *FOXK1* and each of the above genes were calculated using the corr.test function of the R package psych (version 2.4.6.26), and the Benjamini‑Hochberg method was applied for multiple hypothesis testing correction. The significance threshold was set at adjusted P < 0.05 and |cor| > 0.3.

### 2.9. External validation of potential key genes and RT-qPCR validation were performed

To further validate the expression stability of the potential potential key genes, the GSE94019 dataset was downloaded from the GEO database. External validation was conducted using a t-test to compare the expression differences of the potential key genes between the two groups, and the magnitude of the difference between the two groups was assessed based on the absolute value of Cohen’s d. To validate the expression of potential key genes in clinical samples against the bioinformatics predictions, blood samples were collected from 5 healthy controls and 5 DR patients who were recruited from Guangxi Hospital, The First Affiliated Hospital, Sun Yat-sen University, Nanning, Guangxi Zhuang Autonomous Region, China. Clinical information of the study subjects was presented in **S3 Table**. The recruitment period of research subjects was from 20/06/2025 to 31/07/2025. The study was conducted in accordance with the Declaration of Helsinki. This study was approved by the Guangxi Hospital, The First Affiliated Hospital, Sun Yat-sen University, Nanning, Guangxi Zhuang Autonomous Region, China. The approval number and date of approval are as follows: [KY-IIT-2025–008] and [20 June 2025]. All patients provided written informed consent when clinical samples were collected for RT-qPCR experiments to ensure that the research process was in accordance with ethical norms and that the patients’ rights and wishes were fully respected. Total RNA from 10 samples was extracted (Trizol reagent, Vazyme), reverse-transcribed (HP All-in-one kit, Yungeng), and analyzed by RT-qPCR (2x Universal Blue SYBR Green Master Mix, Servicebio) with GAPDH as normalizer. The 2^-ΔΔCt^ method and t-test (p < 0.05) in GraphPad Prism 10 were used for quantification and statistical comparison, respectively. Primer sequences are listed in **S4 Table**.

### 2.10. Statistical analysis

All bioinformatics analyses were performed using R software (v 4.3.3). Inter-group differences were assessed with the Wilcoxon test, considering a p-value < 0.05 as statistically significant.

## 3. Results

### 3.1. Identification of 730 DEGs

**Fig 1:**
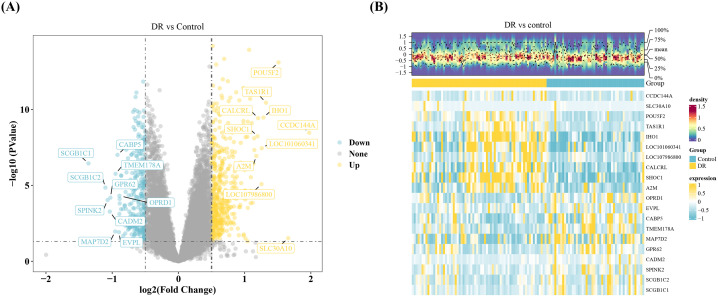
Identification of DEGs in DR (Wald test). **(A)** Volcano plot of DEGs. Yellow dots represent up-regulated DEGs in the DR group, blue dots represent down-regulated DEGs. The top 10 up- and down-regulated DEGs are labeled. **(B)** Heatmap of DEGs. The heatmap displays the expression trends of the top 10 up- and down-regulated genes. Yellow indicates higher expression, blue indicates lower expression.

A total of 730 DEGs (439 upregulated and 291 downregulated) were identified in DR blood samples compared to controls, based on thresholds of p < 0.05 and |log2FC| > 0.5 (Fig 1A-B) (**S5 Table**).

### 3.2. Acquisition of 103 candidate genes

**Fig 2:**
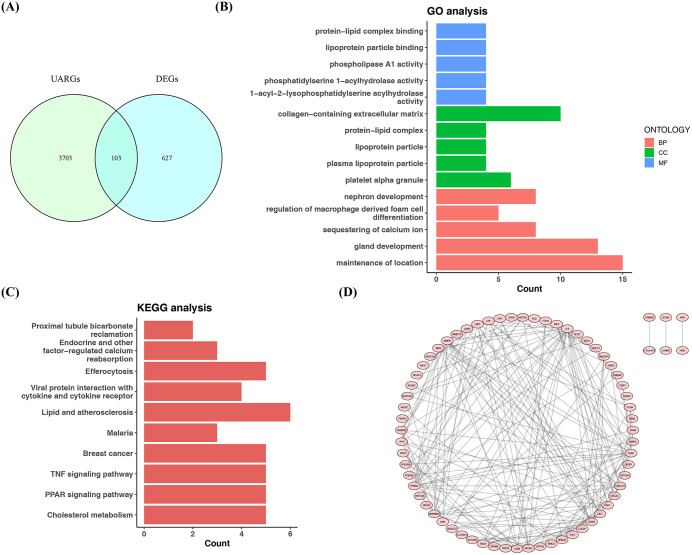
Acquisition and functional annotation of candidate genes. **(A)** Venn diagram showing the candidate genes. **(B)** GO enrichment results. The y-axis labels the GO terms, and the x-axis represents the number of genes enriched in the corresponding term (Hypergeometric test). **(C)** KEGG enrichment results (Hypergeometric test). **(D)** PPI network. Red circles represent proteins, and connecting lines represent interactions between proteins.

Next, the intersection of 730 DEGs and 3,806 UARGs was taken, and a total of 103 candidate genes related to UA in DR were obtained for subsequent analysis **(Fig 2A**). GO enrichment analyses showed that 103 candidate genes were enriched in 465 GO signaling pathways (p-adjust < 0.05). Among them, there were 436 BP pathways, including nephron development, regulation of macrophage derived foam cell differentiation, etc. There were 11 CC pathways, such as collagen-containing extracellular matrix, and 18 MF pathways, such as protein-lipid complex binding **(Fig 2B) (****S6 Table**). These genes were enriched in 186 KEGG signaling pathways (p-adjust < 0.05), such as proximal tubule bicarbonate reclamation **(Fig 2C) (****S7 Table****).**

A PPI network with 69 nodes and 193 edges (interaction score > 0.4) was subsequently constructed from the candidate genes. These interactions included the interaction between *MMP15* and MMP9, as well as between *HCFC1* and *FOXK1*, etc. **(Fig 2D**).

### 3.3. Identification of 2 potential key genes

**Fig 3:**
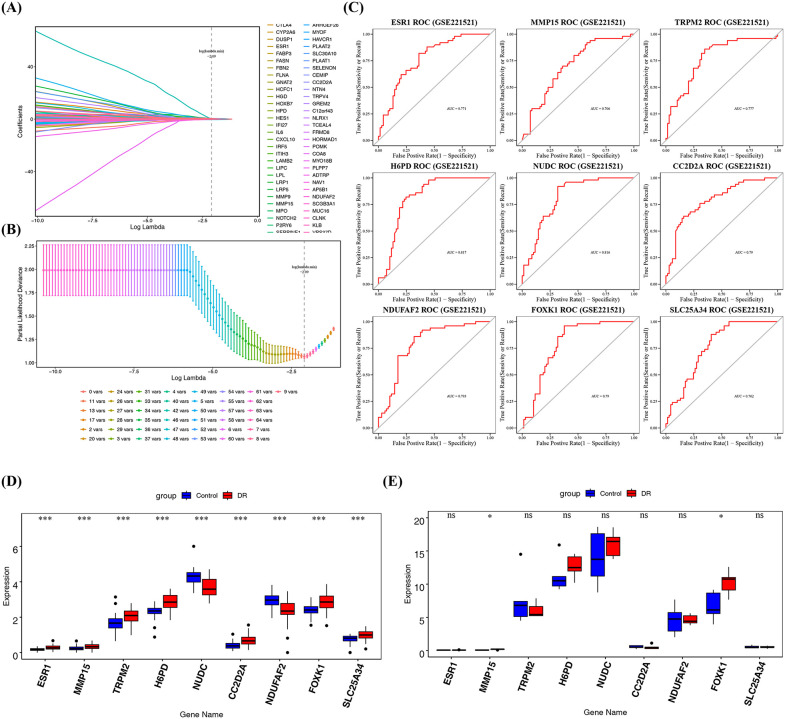
Screening of feature genes and identification of potential key genes. **(A)** LASSO coefficient profile of candidate genes. Each curve represents a gene. The x-axis is the log(λ), and the y-axis is the coefficient. **(B)** Optimal lambda (λ) selection in the LASSO model. **(C)** ROC curves (GSE221521 dataset). A red curve above the grey line with an AUC value greater than 0.7 indicates good classification performance. **(D-E)** Expression levels for screening biomarkers (T test). **(D)** GSE221521 dataset, **(E)** GSE185011 dataset. ns, not significant; *, p < 0.05; **, p < 0.01; ***, p < 0.001.

Using the Lasso algorithm, the optimal λ (log(lambda.min)) value was −2.09, and a total of 9 feature genes were screened, namely *ESR1*, *MMP15*, *TRPM2*, *H6PD*, *NUDC*, *CC2D2A*, *NDUFAF2*, *FOXK1*, and *SLC25A34* (Fig 3A-B)**.**

Subsequently, ROC analysis of the nine feature genes in DR versus control samples revealed that all exhibited AUC values greater than 0.7. Such as *ESR1* had an AUC of 0.771, and *FOXK1* had an AUC of 0.79. These results demonstrated that these feature genes exhibited moderate discriminatory ability and could distinguish DR from control blood samples to some extent **(Fig 3C).**

Finally, Wilcoxon test analysis confirmed significant differential expression for all nine feature genes in the GSE221521 dataset (p < 0.001), following validation across the GSE221521 and GSE185011 datasets **(Fig 3D**). By contrast, significant differential expression in the GSE185011 dataset was limited to two genes, *MMP15* and *FOXK1* (p < 0.05) **(Fig 3E**), and their expression trends were consistent with those in GSE221521. Both genes were significantly upregulated. Therefore, *MMP15* and *FOXK1* were identified as Potential key genes. Further Cohen’s d effect size analysis on the validation dataset showed that the effect sizes of the differences between the two groups for *MMP15* (Cohen’s d = −1.57) and *FOXK1* (Cohen’s d = −1.74) were both at a “very large” level, which to some extent demonstrated the generalizability of the results.

### 3.4. Biological pathways of 2 potential key genes

**Fig 4:**
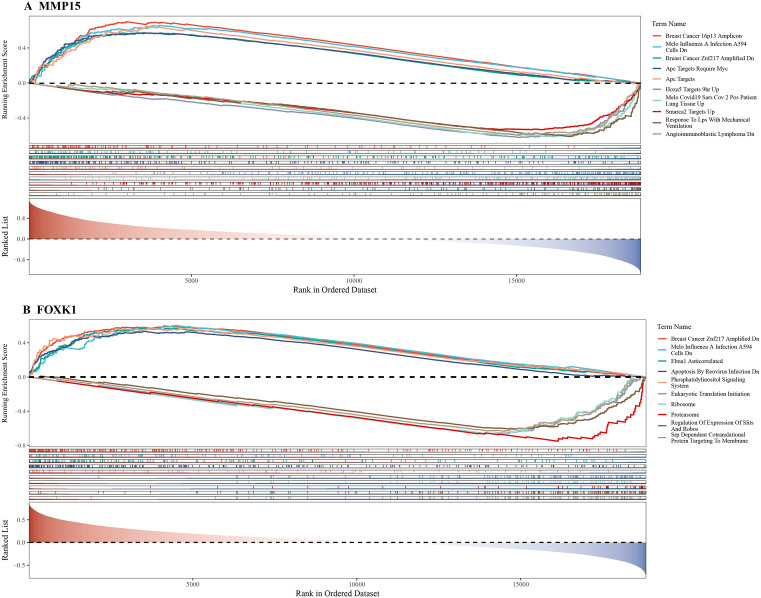
GSEA enrichment analysis (Hypergeometric test).

GSEA showed that *MMP15* was enriched in a total of 740 pathways, such as breast cancer 16p13 amplicon, Apc targets require Myc, and Smarca2 targets up. *FOXK1* was enriched in 833 pathways, including EBNA1 anticorrelated, phosphatidylinositol signaling system, and proteasome (p < 0.05) **(Fig 4) (****S8 Table**). These results suggested that these pathways might be associated with DR.

### 3.5. Immune microenvironment of 2 potential key genes

**Fig 5:**
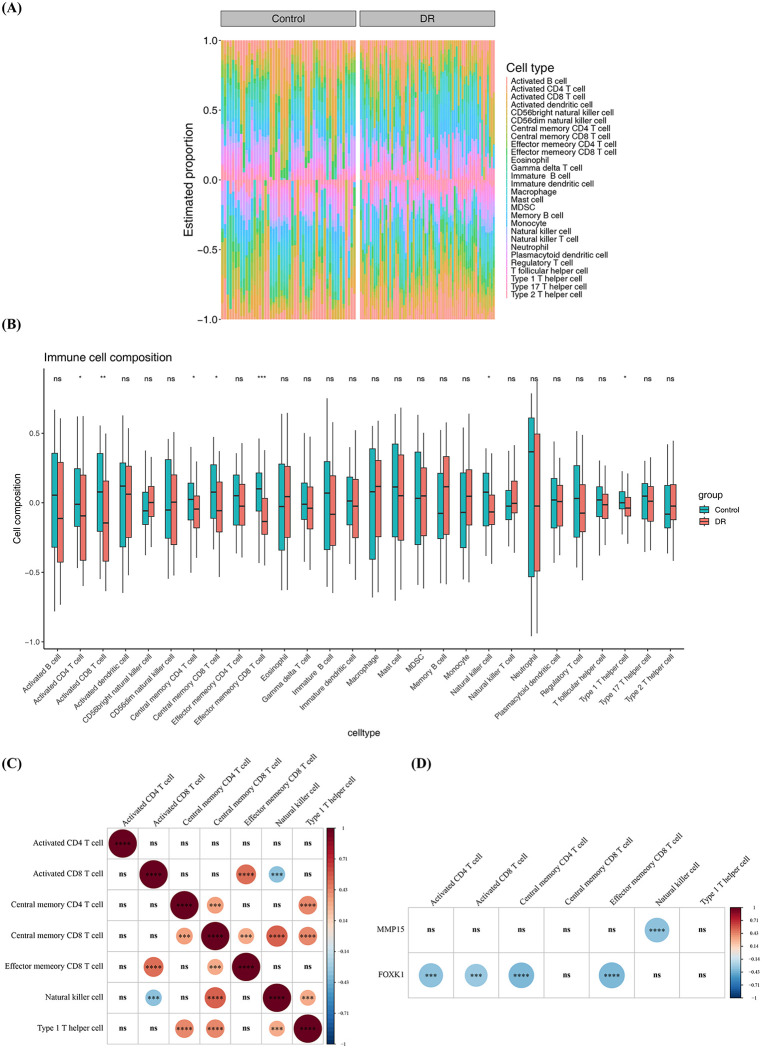
Analysis of immune infiltration. **(A)** Relative proportions of immune infiltration in the DR group vs. the Control group. Different colors represent the ssGSEA scores for various immune cells. **(B)** Differences in immune cell infiltration proportions between the DR group and the Control group. **(C)** Correlations among differentially infiltrated immune cells. Blue indicates negative correlation, red indicates positive correlation (wilcox test). **(D)** Correlation between biomarkers and differentially infiltrated immune cells. ns, not significant; *, p < 0.05; **, p < 0.01; ***, p < 0.001; ****, p < 0.0001 (wilcox test).

Assessment of 28 immune cell types in the GSE221521 dataset (Fig 5A) identified 7 with significantly altered infiltration (Wilcoxon test, p < 0.05), including downregulated activated CD4 and CD8 T cells in DR (Fig 5B). A separate correlation analysis highlighted a strong positive interaction between central memory CD8 T cells and natural killer cells (cor = 0.55, p = 6.23e-11) (Fig 5C) (S9 Table) (S10 Table). In addition, a statistically significant negative association was detected between *MMP15* and natural killer cells (cor = −0.35, p = 9.50e-05), and *FOXK1* was significantly negatively correlated with central memory CD4 T cells (cor = −0.38, p = 2.05e-05) and effector memory CD8 T cells (cor = −0.39, p = 1.24e-05). (Fig 5D) (S11 Table) (S12 Table). These findings suggested that *FOXK1* might be associated with the infiltration levels of central memory CD4 T cells and effector memory CD8 T cells, and might participate in the disease process of DR.

### 3.6. GeneMANIA of 2 potential key genes

**Fig 6:**
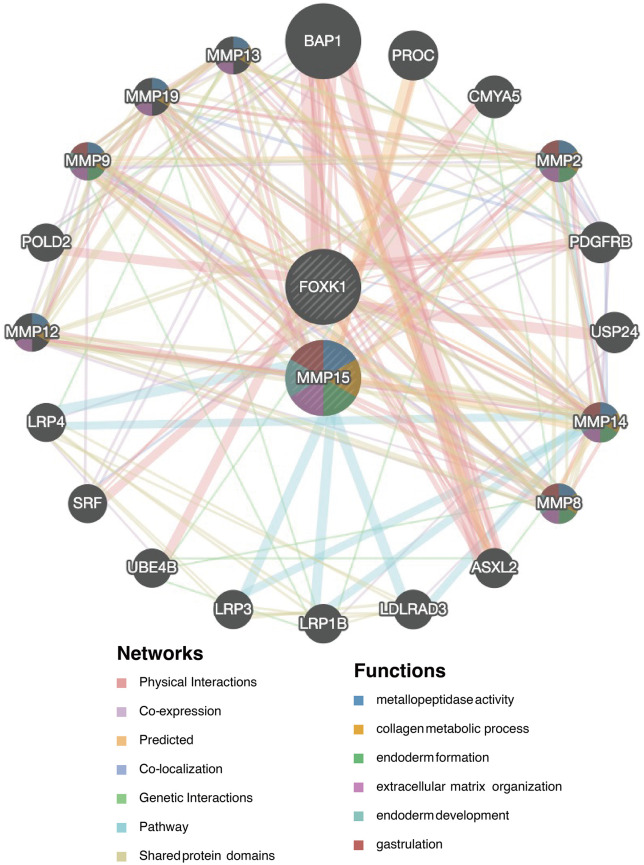
GeneMANIA analysis.

The interaction network among *MMP15*, *FOXK1*, and other related genes was constructed using the GeneMANIA database. The results showed that the relationships among *MMP15*, *FOXK1*, and other genes primarily involved physical interactions, shared protein domains, and pathways **(Fig 6**).

### 3.7. Regulatory network of 2 potential key genes

**Fig 7:**
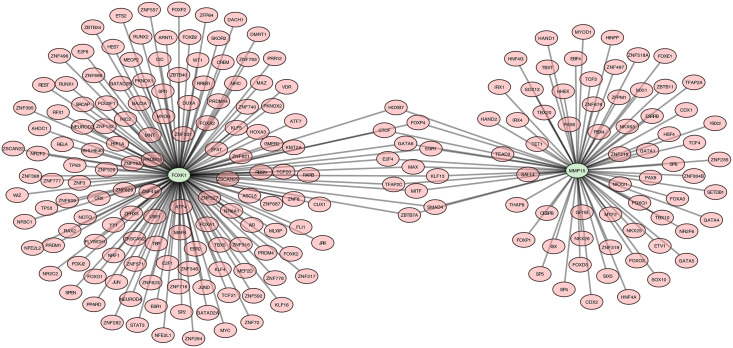
mRNA-TF regulatory network. Green nodes represent biomarker genes, red nodes represent TFs.

An mRNA-TF network with 214 nodes and 224 edges was constructed. 76 TFs targeted *MMP15*, and 148 TFs targeted *FOXK1*. Among these, a total of 12 TFs were found to co-regulate both potential key genes, including HOXB7 and GATA6 targeting both *MMP15* and *FOXK1* **(Fig 7**). At the transcriptional level, these TFs, which had a regulatory effect on *MMP15* and *FOXK1*, may influence the development of DR.

### 3.8. Correlation analysis between FOXK1 and purine metabolism-related genes was performed

To investigate whether *FOXK1* exhibited co‑expression relationships with genes in the purine metabolism pathway, the expression correlations between *FOXK1* and eight purine metabolism‑related genes in the GSE221521 dataset were analyzed. The Pearson correlation analysis revealed two significant correlation pairs: *FOXK1* was significantly positively correlated with IMPDH1 (cor = 0.3, P = 0.00091) and significantly negatively correlated with ATIC (cor = −0.3, P = 0.00079). These results suggested that *FOXK1* might participate in the purine metabolism process by regulating the expression of IMPDH1 and ATIC, thereby affecting uric acid levels (**S1 Fig**).

### 3.9. High expression of *MMP15* and *FOXK1* in DR patient blood samples

To further validate the reliability of the two screened potential key genes (*MMP15* and *FOXK1*), an independent dataset GSE94019 was analyzed in this study. The results showed that, consistent with the findings in the training set, the expression levels of both genes were significantly higher in DR samples than in control samples (p < 0.05), and their expression trends were consistent with those observed in the GSE221521 and GSE185011 datasets (**S2 Fig**). Furthermore, Cohen’s d effect size analysis indicated that the effect sizes of the differences between the two groups for *MMP15* (Cohen’s d = −1.34) and *FOXK1* (Cohen’s d = −2.13) were both at a “very large” level, suggesting that the observed differences were not only statistically significant but also of substantial practical significance.

**Fig 8:**
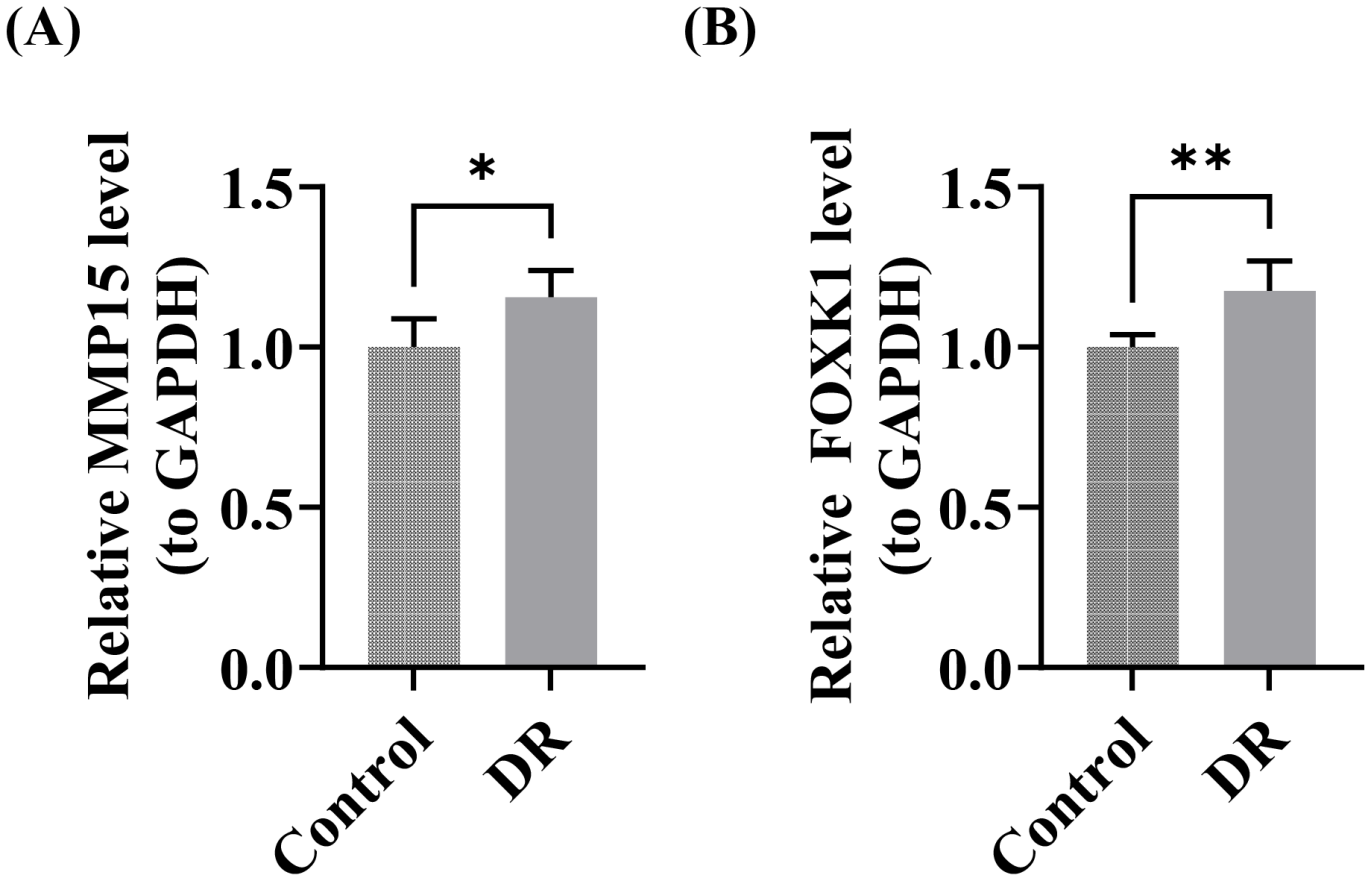
RT-qPCR validation results. **(A)** *MMP15*, **(B)** *FOXK1*. *, p < 0.05; **, p < 0.01.

Clinical samples were used for RT-qPCR validation of *MMP15* and *FOXK1*. The results confirmed that *MMP15* and *FOXK1* were significantly upregulated in DR patient blood samples (p < 0.05) (Fig 8A-B). In summary, the concordance between RT-qPCR results and bioinformatics data collectively verified key gene expression patterns in DR. These findings suggested that the expression patterns of the key genes were reproducible in clinical samples and provided a reference for further investigation of their roles in DR.

## 4. Discussion

In the present study, we identified *MMP15* and *FOXK1* as potential uric acid–related potential key genes in DR through machine learning–based screening, ROC curve evaluation, and expression validation. Both genes were significantly upregulated in DR samples and exhibited moderate-to-high discriminatory ability (AUC > 0.7). Functional enrichment analysis indicated that both genes were enriched in pathways implicated in the pathogenesis of DR. Immune infiltration analysis revealed imbalanced infiltration of multiple immune cell subsets in DR patients, in which *MMP15* and *FOXK1* were negatively correlated with natural killer cells and memory T cells, respectively. Regulatory network analysis identified 12 transcription factors that could co-target these two potential key genes. RT-qPCR validation confirmed elevated expression levels of *MMP15* and *FOXK1* in the peripheral blood of DR patients, supporting their potential value as diagnostic biomarkers.

*MMP15* (MT2‑MMP) is a membrane‑anchored matrix metalloproteinase capable of degrading the extracellular matrix (ECM) and regulating growth factor signaling [31]. These characteristics are consistent with the pathological alterations in retinal vascular diseases: *MMP15* expression is upregulated in the vitreous humor of patients with proliferative diabetic retinopathy (PDR) and is thought to contribute to disease progression [32]. Ranibizumab, an anti‑VEGF agent, can reshape the systemic expression pattern of MMP‑ and TIMP‑related genes in patients with neovascular age‑related macular degeneration, and *MMP15* has been proposed to be potentially involved in this therapeutic response [33]. Moreover, *MMP15* has been identified as a hub gene in glaucoma, suggesting its broader pathogenic relevance in ocular vascular and neurodegenerative disorders [34]. In the present study, *MMP15* was significantly upregulated in the peripheral blood transcriptome of patients with DR, consistently appeared as a high‑weight feature gene in machine learning‑based screening, and its elevated expression was further confirmed by RT‑qPCR. These findings indicate that *MMP15* represents a detectable and reproducible peripheral blood biomarker with potential translational application value.

According to existing literature, although the roles of MMP‑2 and MMP‑9 in DR have been extensively documented, *MMP15* has not been established as a key molecule linking uric acid to DR. Combined with our enrichment analysis, the expression signature of *MMP15* is closely associated with ECM remodeling, increased endothelial permeability, and transendothelial migration of inflammatory cells — processes that underlie early blood‑retinal barrier breakdown, exudation, and immune cell infiltration into retinal tissue in DR [31,32,35]. We hypothesize that the inflammatory microenvironment induced by elevated uric acid may upregulate *MMP15* expression [36,37]. As a membrane‑type matrix metalloproteinase, *MMP15* can activate downstream MMP‑2 [38], and this cascade may constitute a potential molecular pathway connecting uric acid metabolic disturbance to DR vasculopathy.

*FOXK1* (Forkhead Box K1) is a member of the forkhead box transcription factor family and participates in key cellular processes including cell cycle regulation, DNA damage response, metabolic reprogramming, angiogenesis, and apoptosis [39,40]. The VEGF/VEGFC signaling axis serves as a core driver of pathological angiogenesis and vascular leakage in DR [41]. Recent evidence indicates that upregulation of *FOXK1* enhances VEGFC expression [42]. Conversely, inhibition of *FOXK1* suppresses the PI3K/Akt/mTOR pathway and subsequent cell proliferation and angiogenesis [43]. These findings position *FOXK1* as a relevant regulator at the interface between metabolism and vascular remodeling. Notably, *FOXK1* also exhibits similar pathogenic roles in other microvascular complications. For instance, *FOXK1* knockdown has been shown to alleviate glomerular endothelial injury and renal fibrosis in mice [44]. In the present study, *FOXK1* was consistently upregulated in two independent peripheral blood transcriptome datasets of DR patients, and its elevated expression was further validated by RT‑qPCR. In addition, correlation analysis revealed that *FOXK1* was significantly positively correlated with IMPDH1, a key enzyme in purine nucleotide synthesis, while negatively correlated with ATIC, which participates in purine nucleotide interconversion. This suggests a potential regulatory link between *FOXK1* and purine metabolism at the transcriptional level. Collectively, these findings imply that *FOXK1* may participate in metabolic reprogramming and angiogenesis regulation in DR patients. This hypothesis warrants further mechanistic investigation for validation.

Regarding pathway enrichment analysis, the Gene Set Enrichment Analysis (GSEA) results for *MMP15* and *FOXK1* revealed distinct functional orientations. The gene sets associated with *MMP15* were enriched in the lipopolysaccharide (LPS) response pathway, which is linked to the activation of innate immune cells. Previous studies have demonstrated that LPS can affect the function of retinal microvascular endothelial cells by activating the TLR4 signaling pathway [45], suggesting that *MMP15* may be associated with the innate immune response and thereby indirectly participate in vascular lesions in DR. In contrast, the gene sets related to *FOXK1* were enriched in the phosphatidylinositol signaling system and proteasome pathways. As an upstream regulatory component of the PI3K/Akt signaling axis, the phosphatidylinositol signaling system and its associated axis have been confirmed to be involved in DR-related pathological angiogenesis [46]. The proteasome pathway, in turn, is involved in maintaining intracellular protein homeostasis [47]. As a metabolism-related transcription factor [40], the enrichment profile of *FOXK1* indicates that this gene may participate in the formation of retinal neovascularization during the progression of DR by influencing intracellular metabolic signal transduction and protein homeostasis. It should be emphasized that the above speculations are mainly based on indirect evidence from existing literature and require further functional experiments for validation.

In the present study, we observed a negative correlation between *MMP15* expression and natural killer [48] cell infiltration level. NK cells are thought to exert potential protective functions in DR [49]. As a membrane-type matrix metalloproteinase, *MMP15* participates in extracellular matrix remodeling and the processing and release of inflammatory factors [50]. Combined with the above evidence, we hypothesize that high *MMP15* expression may indirectly modulate the recruitment or activity of NK cells by shaping the local inflammatory cytokine network, thereby attenuating their potential protective effects. This hypothesis provides a plausible molecular entry point for understanding the involvement of *MMP15* in immune regulation in DR.In addition, *FOXK1* expression was negatively correlated with the infiltration levels of central memory CD4 ⁺ T cells and effector memory CD8 ⁺ T cells. As a key transcription factor involved in metabolic reprogramming and cellular stress responses, *FOXK1* contributes to insulin regulation and mitochondrial-dependent cellular metabolism and function [40]. The differentiation, maintenance, and migratory capacity of memory T cells are highly dependent on metabolic status [51]. We therefore speculate that elevated *FOXK1* expression may alter the metabolic fitness of T cells, thereby affecting the distribution or survival of specific memory T-cell subsets in peripheral blood and further contributing to DR-related systemic immune alterations. However, the above speculations are mainly based on statistical associations and indirect evidence from existing literature, and it remains unclear whether these immune changes act as drivers of DR progression or are concomitant phenomena secondary to systemic metabolic disorders. Future studies incorporating retinal tissue samples, longitudinal cohorts, and functional experiments are warranted to further clarify the specific roles of *MMP15* and *FOXK1* in immune regulation and their association with the pathological mechanisms ‌‌of DR.

Of particular note, as a local disease centered on retinal microvascular lesions, ideal molecular biomarker studies for DR should ideally be derived directly from ocular tissue. However, numerous recent studies have demonstrated that DR is not an isolated ocular disorder, but is associated with systemic metabolic disturbances, chronic low-grade inflammation, and imbalanced immune status [52]. Moreover, an increasing number of studies have employed peripheral blood samples to identify potential key genes or biomarkers related to DR, which have shown favorable diagnostic and risk-prediction value [53,54]. Therefore, the use of peripheral blood data for biomarker discovery offers unique advantages in clinical translation, including noninvasiveness, easy accessibility, and convenience for dynamic monitoring. Nevertheless, we acknowledge that peripheral blood gene expression profiles may not fully and precisely reflect local molecular events in the retina. The precise localization, expression changes, and functional mechanisms of the currently identified potential key genes in retinal tissue still require further experimental validation. Accordingly, in future studies we will combine animal models and retinal tissue specimens to further investigate the exact roles of the screened potential key genes in local ocular lesions, with the aim of more comprehensively elucidating their pathological significance.

Taken together, our findings support a preliminary working model: uric acid-driven chronic low-grade inflammation and metabolic stress may be linked to *MMP15*-mediated alterations in vascular permeability and *FOXK1*-related PI3K/Akt signaling, which collectively contribute to the pathogenesis of DR. This model warrants further validation by functional experiments. Nevertheless, several limitations should be acknowledged. The sample size remains relatively small, and experimental validation is currently restricted to peripheral blood. We lack direct functional evidence from single-cell spatial resolution, metabolic profiling, as well as models including endothelial barrier assays, angiogenesis assays, and in vivo intervention studies. Furthermore, due to missing information, the included samples lacked comparisons of key clinical characteristics such as diabetes duration, DR stage, blood glucose, and uric acid levels, which may introduce certain bias in interpreting gene expression differences. Therefore, the precise causal positioning of *MMP15* and *FOXK1* in the DR pathological cascade remains to be determined. Future work will focus on expanding the sample size and recruiting large independent cohorts with complete clinical information, to further clarify the independent expression regulatory relationships of *MMP15* and *FOXK1* in DR by adjusting for confounders through multivariate analysis. Meanwhile, future studies will determine whether *MMP15* directly regulates blood‑retinal barrier permeability, whether *FOXK1* amplifies pathological neovascularization via the PI3K/Akt/mTOR pathway, and whether these two genes form a synergistic regulatory loop under uric acid-driven chronic inflammation. Addressing these issues may provide a molecular basis for early risk stratification, progression control, and individualized intervention in DR.

## Supporting information

S1 Table Clinical Information Table of the Dataset. (XLSX)

S2 Table List of UARGs from GeneCards. (XLSX)

S3 Table Clinical Sample Clinical Information Form. (DOCX)

S4 Table Primer sequences used for RT-qPCR validation of Potential key genes. (XLS)

S5 Table DEGs identified in the GSE221521 dataset. (XLSX)

S6 Table GO enrichment analysis of candidate genes. (XLSX)

S7 Table KEGG pathway enrichment analysis of candidate genes. (XLSX)

S8 Table GSEA results for Potential key genes. (XLSX)

S9 Table Correlation matrix of differentially infiltrated immune cell types in the GSE221521 dataset. (XLSX)

S10 Table P-value matrix for immune cell type correlations in the GSE221521 dataset. (XLSX)

S11 Table Correlation coefficients between potential key genes and differentially infiltrated immune cells. (XLSX)

S12 Table P-values for correlations between potential key genes and immune cells. (XLSX)

S1 Figure Analysis results of the correlation between *FOXK1* and genes related to purine metabolism. (TIF)

S2 Figure Validation of potential key gene expressions in the GSE94019 dataset. (TIF)

## Abbreviation:

DR: diabetic retinopathy
UA: uric acid
UARGs: UA-related genes
ROC: receiver operating characteristic
GSEA: gene set enrichment analysis
AUC: area under the curve
RT-qPCR: reverse transcription quantitative polymerase chain reaction
TF: transcription factor
NPDR: non-proliferative diabetic retinopathy
PDR: proliferative diabetic retinopathy
GEO: Gene Expression Omnibus
PRP: anretinal photocoagulation
TNF-α: Tumor necrosis factor-α
IL-6: Interleukin-6
CRP: C-reactive protein
DEGs: differentially expressed genes
FC: fold change
GO: Gene Ontology
KEGG: Kyoto Encyclopedia of Genes and Genomes
LASSO: least absolute shrinkage and selection operator
ssGSEA: single-sample gene set enrichment analysis
PPI: protein-protein interaction
DN: diabetic nephropathy
DPN: diabetic peripheral neuropathy
ECM: extracellular matrix
LPS: lipopolysaccharide
Tem: Effector memory T cells
NKT: natural killer T
NK: natural killer
