# Supplementary material for: Transcriptome sequencing combined with experimental verification to explore potential key genes related to uric acid in diabetic retinopathy
Source: PLoS One. 2026 Jun 2;21(6):e0350132. doi: 10.1371/journal.pone.0350132 (PMC13229317; doi:10.1371/journal.pone.0350132)
Supplement: S3 Table — (DOCX) [file pone.0350132.s003.docx]

S3 Table Clinical characteristics statistics of DR clinical samples

| **No.** | **Gender** | **Duration of diabetes (years)** | **DR stage** | **HbA1c (%)** | **Uric acid (μmol/L)** | **Group** |
| --- | --- | --- | --- | --- | --- | --- |
| 1 | Male | 5 | PDR | 8.2 | 442 | Diabetic retinopathy |
| 2 | Male | 6 | PDR | 5.2 | 433 | Diabetic retinopathy |
| 3 | Male | 5 | PDR | 10.2 | 483 | Diabetic retinopathy |
| 4 | Female | 13 | NPDR | 6.56 | 275 | Diabetic retinopathy |
| 5 | Female | 12 | NPDR | 6.50 | 194 | Diabetic retinopathy |
| 6 | Female | N/A | N/A | N/A | N/A | Control |
| 7 | Female | N/A | N/A | N/A | N/A | Control |
| 8 | Female | N/A | N/A | N/A | N/A | Control |
| 9 | Female | N/A | N/A | N/A | N/A | Control |
| 10 | Female | N/A | N/A | N/A | N/A | Control |
