# Supplementary figures and images for: Transcriptome sequencing combined with experimental verification to explore potential key genes related to uric acid in diabetic retinopathy
Source: PLoS One. 2026 Jun 2;21(6):e0350132. doi: 10.1371/journal.pone.0350132 (PMC13229317; doi:10.1371/journal.pone.0350132)

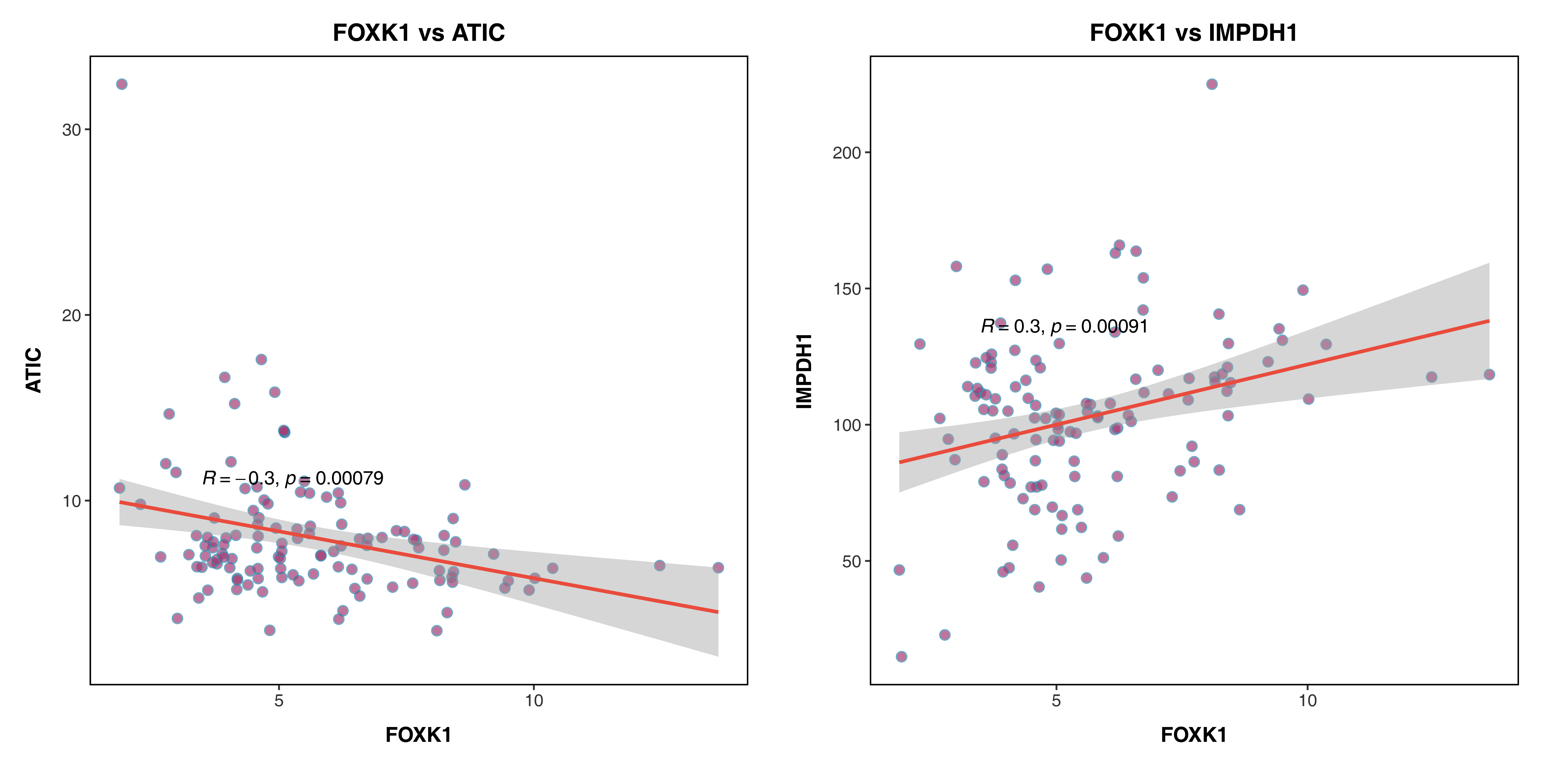

Supplement: S1 Figure — (TIF) [file pone.0350132.s013.tif]

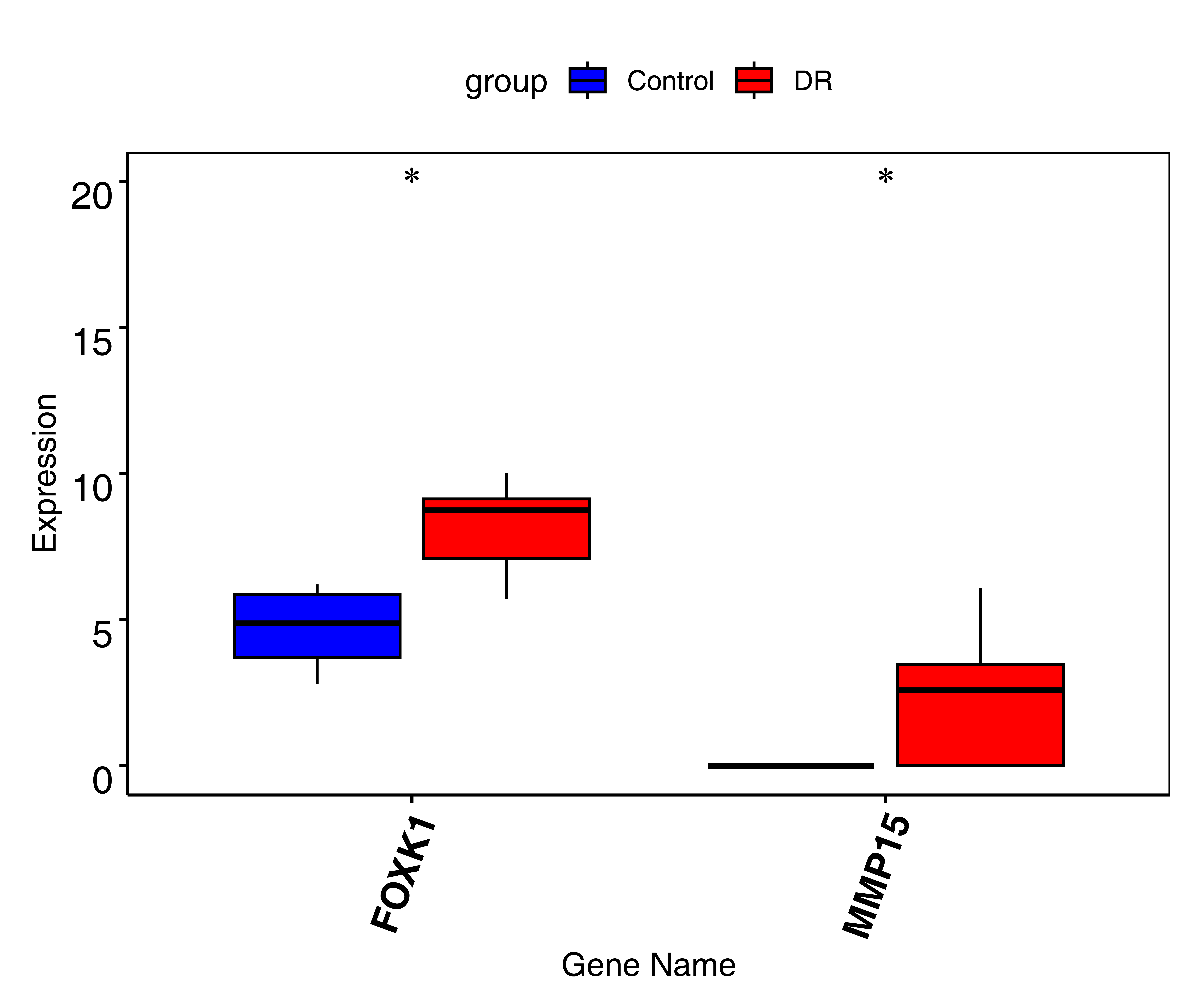

Supplement: S2 Figure — (TIF) [file pone.0350132.s014.tif]
